# Anti-VEGF therapies for age-related macular degeneration: a powerful tactical gear or a blunt weapon? The choice is ours

**DOI:** 10.1007/s00417-021-05451-2

**Published:** 2021-10-20

**Authors:** Paolo Lanzetta

**Affiliations:** 1 Department of Medicine - Ophthalmology, University of Udine, Udine, Italy; 2 Istituto Europeo di Microchirurgia Oculare - IEMO, Via M.A. Fiducio, 8, 33100 Udine, Italy

**Keywords:** Blindness, Visual impairment, Vision loss, Age-related macular degeneration, Anti-VEGF, Health care model

## Abstract

**Purpose:**

Blindness and vision loss are still frequent disabilities associated with a relevant impact on health care and quality of life, and a high economic burden. Supranational programs established by the World Health Organization (WHO), International Agency for the Prevention of Blindness (IAPB), and World Health Assembly (WHA) aim at reducing avoidable visual impairment. Age-related macular degeneration (AMD), diabetic retinopathy (DR), and other retinal diseases are well known causes of visual disability. Since more than a decade, intravitreal agents are available for the treatment of these diseases. The aim of this study is to review whether pharmacotherapy with anti-vascular endothelial growth factor (VEGF) drugs has led to a decrease in the prevalence of blindness with emphasis on AMD and different countries. A brief analysis of other factors correlated to changes in the rate of blindness is also presented.

**Methods:**

PubMed and Scopus web platforms were used to identify relevant studies on epidemiology of blindness and vision impairment, the influence of intravitreal therapies, and the existence of different vision care models. Additional data and material was searched in web internet accessed by the web browser Firefox.

**Results:**

Age-standardized prevalence of blindness secondary to AMD has started to decline as testified by a number of studies in different countries. This is due to the adoption of anti-VEGF therapy and its adequate management. The frequency of treatment and regimens applied are indirect signs of successful treatment. Local rules and regulations may represent an obstacle.

**Conclusions:**

This review shows that by implementing existing health care systems and dispensing adequate therapies in the field of retinal diseases, the prevalence of blindness due to these conditions can decline.

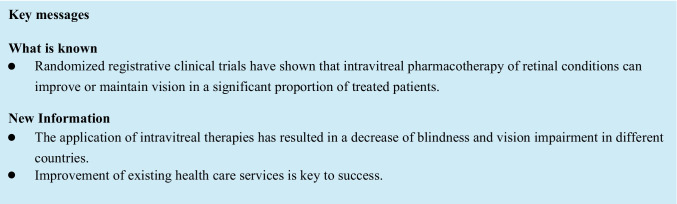

## Introduction

In 1999, the WHA and IAPB developed a project called “Vision 2020: The Right to Sight” whose aim was to eradicate avoidable blindness [1]. The initiative is based on three mainstays: disease control, human resource development, and infrastructure development [2].

In 2013, the WHO promoted a new plan, “Towards universal eye health: a global action plan 2014–2019 (GAP).” It identifies a global aim: to reach by 2019 a 25% reduction from the baseline of 2010 in prevalence of avoidable visual impairment. The premiss is that effective and accessible eye care services are key for successfully controlling visual impairment including blindness. Similarly to Vision 2020, GAP is built on principles and approaches: universal access and equity, human rights, evidence-based practice, a life course approach, and empowerment of people with visual impairment. The efficacy of the plan is also dependent on the development of comprehensive health systems and human resources for health development [3].

Therefore, both Vision 2020 and GAP emphasize that developing human and technological resources and reorganizing health systems are key to success.

In 2015, it was estimated that 36 million people were blind, 217 million were moderately or severely vision impaired, and 188 million had mild vision impairment [4].

Now, age-related macular degeneration (AMD) is a leading cause of vision impairment and legal blindness in elder subjects. Due to the aging population, the global prevalence of AMD is expected to increase from 170 to 288 million by the year 2040 [5].

A recent publication on the causes of blindness and vision impairment found that the age-standardized prevalence of blindness due to AMD declined by almost 30% from 1990 to 2020. This decrease is likely due to the widespread clinical introduction of anti-VEGF therapy for this condition [1].

The aim of this study is to review whether pharmacotherapy with anti-VEGF agents had an influence on the prevalence of blindness with emphasis on AMD and different countries. A search of other influencing factors has also been performed.

## Methods

PubMed and Scopus web platforms were used to identify relevant studies on epidemiology of blindness and vision impairment, the influence of intravitreal therapies, and the existence of different strategies and vision care models. The following keywords were used: blindness, visual impairment, vision loss, age-related macular degeneration, anti-VEGF, health care model.

Additional data and material was searched in web internet accessed by the web browser Firefox.

## Results

Relevant published experiences with significant information are summarized in Table 1.

**Table 1:** Summary of relevant selected studies

Source	Country	Relevant information
Bressler NM et al. Arch Ophthalmol 2011 [6]	USA	Modeling on monthly ranibizumab/24 months: 2-year incidence of legal blindness − 72%, visual impairment − 37%
Skaat A et al. AJO 2012 [7]	Israel	Rate of blindness: 1999: 33.8/100000; 2008: 16.6/100000. − 51%
Bloch SB et al. AJO 2012 [8]	Denmark	Rate of blindness due to AMD: 2000: 52.2/100000; 2010: 25.7/100000. − 50%
Belkin M et al. AJO 2013 [9]	Israel	Rate of blindness: 2008: 16.6/100000; 2010: 14.8/100000
		Due to AMD: 2008: 3.51/10000; 2010: 2.84/100000
Borooah S et al. Eye 2015 [10]	Scotland	Incidence of legal blindness: 2006: 9.1/100000; 2011: 4.8/100000. − 47%
Rim TH et al. Clin Exp Ophthalmol 2017 [11]	South Korea	Incidence of blindness: 2002: 43.8/100000; 2013: 15.9/100000
Claessen et al. Graefes Arch Clin Exp Ophthalmol 2021 [12]	Saxony	Rate of blindness: 2009: 15.7/100000; 2017: 8.9/100000
		Due to AMD: 2009: 6.9/100000; 2017: 3.8/100000
		Due to DR: 2009: 1.5/100000; 2017: 0.7/100000
Heath Jeffery RC et al. Asia Pac J Ophthalmol 2021 [13]	Australia	Age-standardized annual incidence of blindness rose during the PDT era, reaching 72.5 cases per 100,000 person-years in 2004. The incidence declined from 2007 onwards, reaching 8.2 cases per 100,000 person-years in 2016 (IVT era)
Holz FG et al. BJO 2015 [14]	Multiple	Mean of 5.0 and 2.2 injections in the first and second year (Ireland 11, Canada 9.9, the UK 9, The Netherlands 8.7, France 6.3, Germany 5.6, Italy 5.2, Venezuela 3.2)

*AMD*, age-related macular degeneration; *DR*, diabetic retinopathy

Before observational epidemiological studies were available, a modeling on non-Hispanic white population in the USA estimated that the use of monthly ranibizumab for 24 months would reduce the 2-year incidence of legal blindness by 72% and of visual impairment by 37% [6].

Subsequently, according to two studies, in Israel there was a continuous decrease in the total annual age-standardized rate of blindness certification per 100,000 residents, from 33.8 in 1999 to 16.6 in 2008. This is a drop of 51% throughout the decade [7]. A further analysis for the years 2009 and 2010 showed the same trend with a continuation of the rapid decline in the incidence of new cases of blindness from all major causes except cataract. The overall rate of blindness per 100,000 people decreased from 16.6 in 2008 to 14.8 in 2010. For AMD, the rates for the corresponding years were 3.51 and 2.84, respectively [9].

In Denmark, the incidence rate of legal blindness secondary to AMD declined from 52.2 cases per year per 100,000 citizens in 2000 to 25.7 cases per year per 100,000 in 2010, corresponding to a decline of 50% [8].

In Scotland, the age-sex standardized incidence of legal blindness changed from 9.1 cases per 100,000 in 2006 to 4.8 in 2011 following the introduction of intravitreal ranibizumab treatment. This is a drop of 47% from the peak level [10].

Similarly, in South Korea the incidence of blindness between 2002 and 2013 dropped from 43.8 per 100,000 person-years to 15.9 [11].

Claessen et al. observed a significant decline in the incidence of blindness between 2009 and 2017 in Eastern Germany. All causes of blindness decreased from 15.7 per 100,000 person-years to 8.9. In terms of specific diseases, the incidence decreased for AMD (2009: 6.9, 2017: 3.8), diabetic retinopathy (DR) (2009: 1.5, 2017: 0.7), and other conditions [12]. Similar tendencies in Germany were previously reported with the highest positive trends for AMD [15, 16].

A recent publication reports the age-standardized annual incidence of blindness registration due to AMD in Australia in patients aged 50 years and older. Frequencies of photodynamic therapy (PDT) and intravitreal therapy were examined over a 21-year period (1996–2016). In a population residing in Western Australia where a uniform treatment strategy for neovascular AMD was available, the percentage of reported blindness due to AMD peaked at 77% in 2006 and declined to 41% by 2016. Although the authors admit that no conclusive evidence on the effectiveness of intravitreal therapies can be drawn due to limitations on registry data, reported information suggest that a rapid increase in the incidence of blindness due to AMD was registered at the time of PDT and a decline from 2007 onwards was seen when intravitreal therapy replaced PDT as preferred treatment of AMD [13].

The AURA study was a multi-country real-life experience of anti-vascular endothelial growth factor therapy for wet age-related macular degeneration. This retrospective, observational study was performed in Canada, France, Germany, Ireland, Italy, the Netherlands, the UK, and Venezuela. Medical records of patients with wet AMD, who started ranibizumab treatment between 1 January 2009 and 31 August 2009, were evaluated. Data were collected until the end of treatment and/or monitoring or until 31 August 2011. A total of 2227 patients received a mean of 5.0 and 2.2 injections in the first and second year, respectively. There were substantial differences in visual outcomes and injection frequency between countries. More frequent visits and injections were associated with greater improvements in visual acuity. Specifically, in all the countries considered, there was an initial improvement in visual acuity followed by a decrease in the visual acuity gains. This decrease was least pronounced in the UK and the Netherlands, where there remained an improvement in visual acuity between baseline and year 2. In contrast, there was a decline in mean visual acuity from baseline to year 2 in patients in Germany, France, and Italy. Among countries enrolling more than 400 patients, Italy had the lowest mean number of injections over 2 years (5.2) with a decrease in visual acuity score to year 2 of 2.9 letters whereas UK showed an improvement of 4.1 letters [14].

Data from registries in Italy report 116,932 and 122,000 subjects with complete/partial blindness in 2017 and 2018 respectively and 1.5 million people with low vision in 2018 [17, 18].

Interestingly, data on drug consumption in Italy for ocular diseases, including glaucoma, are quite eloquent. Although intravitreal therapies of macular and retinal conditions have become the gold standard and allow a successful treatment, during the last 7 years the consumption has remained stable with a defined daily dose/1000 inhabitants (DDD) of 19.9 in 2014 and 21.1 in 2020 with a mean annual change of 1% [19, 20].

For anti-VEGF agents, per capita expenditure in Italy is EUR 1.5 with a significant 36.9% decrease from 2019 to 2020. The amount was the same in 2014 (EUR 1.5). The compound annual growth rate between 2014 and 2020 was − 0.1%. In 2020, the DDD was 0.3 with a decrease of 28.5% versus 2019. It was 0.1 in 2011 and it increased minimally to 0.2 in 2013 [19, 20].

Data from the Italian Drug Agency (AIFA) registry from February 2013 on 48,484 patients with neovascular AMD show that 25.9% received aflibercept, 26.3% received bevacizumab or its biosimilar, 47.5% received ranibizumab, and 0.3% received pegaptanib [20]. In 2020, bevacizumab has been the most used anti-VEGF agent with 1880 new eyes treated/month followed by aflibercept with 871 eyes and ranibizumab with 568 eyes [20].

Unfortunately, the mean number of anti-VEGF injections during the first year of treatment is very low (3.6; aflibercept 3.8, bevacizumab 3.6, ranibizumab 3.5) [20].

As regards strategies and vision care models which may optimize outcomes from the application of intravitreal pharmacotherapy and ultimately result in a decrease of the rate of blindness and vision impairment, different proposals were found through our search.

In Israel, the relevant achievements reported are related not only to the availability of new technologies and effective therapies but, not less importantly, to the universal free access to modern health care, and changes in reimbursement policy. Moreover, of note is that the demand of novel treatments for AMD and other retinal conditions brought to an increase in the number of ophthalmologists dedicated to intravitreal injections [7, 9].

In Denmark, improvements in public health had a crucial role on the favorable outcomes on vision disabilities [8].

Ciulla et al. applied the Lean Six Sigma approach to improve health care delivery and ophthalmology clinic efficiency. They found that this method can notably improve patient care, minimize waste, and enhance satisfaction in a busy vitreoretinal practice [21].

Amoaku et al. reported that in the UK, after the introduction of anti-VEGF therapy of AMD, there have been substantial improvements in rapid referral and fast-track processes for new patients. However, clinical capacity issues limit efficiency. In order to improve patient management, the authors reviewed some exemplar case studies as possible useful solutions: expanded roles for non-consultant clinical staff, mobile eye care clinic for wet AMD, nurse-led rapid access clinics and virtual review clinics, utilization of a peripheral clinic and mobile screening, flexible multidisciplinary approach to patient management and satellite macular clinics in the community, e-referral link between community optometrists and hospital eye clinics, telemedicine pilot project with OCT image transfer from community optometry to ophthalmologist [22].

## Discussion

Our community is grateful to the pivotal work by Judah Folkmann who in 1971 postulated that tumor growth and development are secondary to recruitment of endothelial cells and then vasculature growth [23]. Subsequently, in the 1980s Napoleone Ferrara and his group led to the application of antiangiogenic therapies in ophthalmology [24].

Soon after the approval of the anti-VEGF agent bevacizumab for the treatment of cancer, pegaptanib (Macugen) was approved by the FDA in 2004 for the treatment of neovascular AMD. A new era had began [25]. In 2005, Rosenfeld and his group published on the off-label intravitreal use of bevacizumab for the treatment of AMD [26]. Later on, a number of other innovative anti-VEGF agents were approved for human and ocular use: ranibizumab (Lucentis), aflibercept (Eylea), conbercept (Lumitin), and more recently brolucizumab (Beovu). Since more than a decade, the use of these novel therapies has allowed a paradigm shift in the prognosis of our patients. Improvement of visual acuity is now considered the gold standard whereas in the near past nonselective laser therapy or photodynamic therapy did generally lead to a decline in vision.

The current and widespread use of anti-VEGF therapy for the treatment of a number of ocular conditions such as AMD and DR has now the potential to decrease the prevalence of blindness and visual impairment. Our search highlighted various reports that testified this trend in different countries. A common finding is that the bulk of the reduction in visual disability occurred after the introduction of intravitreal therapies with anti-VEGF agents in 2006.

Experience gained by retina experts in the use of newer therapies during the last decade has also been pivotal in improving outcomes and reducing visual loss in the treated populations. Changes in the strategy of anti-VEGF regimen in real-life settings have conducted to the application of treat-and-extend approaches rather than pro re nata (PRN) with subsequent reduced burden and similar outcomes to the monthly administration regimens applied in registrative trials [27, 28]. Recently, a Bayesian network meta-analysis on neovascular AMD concluded that the treat-and-extend regimen proved to be the most effective approach for each anti-VEGF drug available [29].

Blindness and visual impairment are a public health problem with a significant impact on health care and quality of life, and a high economic burden [30].

All the reported international experiences indirectly prove that by applying innovative therapies within the context of organized health systems, with appropriate human and technological resources, with adequate surveillance of quality indicators and blindness registries, medicine can have a tremendous impact on the course of serious diseases like neovascular AMD which is associated to visual loss if not promptly and regularly treated.

However, we should not forget that the wet type of AMD represents only a minor proportion of the disease spectrum. The dry type of AMD including geographic atrophy (GA) accounts for 85 to 90% of cases and wet AMD for 10 to 15%. Moreover, GA is frequently aggravating neovascular AMD. Although many potential pharmatherapies are under development for dry AMD and GA, including anti-inflammatory substances, neuroprotective agents, and complement inhibitors, none is currently available [31]. It is likely that future advancements on basic and clinical research of dry AMD and GA will possibly translate into newer therapeutic agents and contribute to further reductions of visual disability.

Despite the significant progresses in the cure of AMD, some countries may not experience improved outcomes as suggested by the AURA study. This may not be related to the frequency of treatment only [14].

Despite the fact that Italy was among the first countries to officially reimburse the off-label and less costly bevacizumab for the treatment of this AMD and that more recently AIFA declared that aflibercept, bevacizumab, brolucizumab, and ranibizumab can be considered same drugs in AMD, these decisions did not translate into major progresses than reported by the AURA study [19].

Many reasons may have contributed to the above described suboptimal scenario. On general, since anti-VEGF therapy was introduced in the early 2000s, the national health care system has not been appropriately reorganized to improve clinic efficiencies. In other countries, besides investments in spaces and technological and human resources, many proposals to render intravitreal pharmacotherapy of macular and retinal conditions a real game changer have been applied and they translated into the decrease of disabilities due to visual loss. Linear clinic models, parallel clinic models, expanded non-consultant roles, nurse-led rapid access and virtual review, satellite macular clinics, peripheral clinics and mobile OCTs, community OCT telemedicine, mobile community eye care clinics, and telemedicine with community imaging are many examples that may improve patient flow and efficiency in the clinic. Obviously, clinic space, staffing, staff skills, equipment, support and quality, management planning, business case development, and funding are all chapters which should be approached and implemented [21, 22].

Also, simple rules regulating the administration of intravitreal therapies would greatly help access to care. In most countries, intravitreal injections are given in outpatient surgery rooms and centers or even in the office as in the case of the USA.

In Italy, obsolete regulations imply that intravitreal agents (classified as OSP) can be administered only inside hospitals or comparable facilities. Unfortunately, none of health authorities has defined yet what is comparable to hospitals for this specific therapeutic activity. More than that, only highly specialized hospitals who have been appointed by each regional government are authorized to dispense therapies with intravitreal agents. Differently, the international community agrees that intravitreal agents do not necessarily require a hospital setting for safe administration as they can be given comfortably, safely, and less costly in an ambulatory extra-hospital environment.

The still ongoing COVID-19 pandemic has further complicated the situation. A recent report on the effect of COVID-19-related lockdown on ophthalmic practice in 39 institutional Italian centers has evidenced that intravitreal injections were reduced by 48.5% and 48.6% in the lockdown period in comparison to intra-year and inter-year control periods, respectively [32].

Access to reimbursed off-label bevacizumab alone is not sufficient to improve the system to efficacy and efficiency. The success of intravitreal therapy programs and services is the result of various stakeholders’ engagement in the whole process as well as the improvement of existing health care systems and facilities, and the reassessment of human and technological resources needed.

In the time of artificial intelligence, home-based OCT monitoring, and even home-administered intravitreal therapies, as done in Israel during the lockdown period [33], it is our duty to do everything possible to protect our patients’ sight.

## Data Availability

Not applicable.
